# Recruitment of PI4KIIIβ to the Golgi by ACBD3 is dependent on an upstream pathway of a SNARE complex and golgins

**DOI:** 10.1091/mbc.E23-09-0376

**Published:** 2023-12-22

**Authors:** Danièle Stalder, Igor Yakunin, Conceição Pereira, Jessica Eden, David C. Gershlick

**Affiliations:** a Cambridge Institute for Medical Research, University of Cambridge, Cambridge CB2 0XY, United Kingdom; b MRC Laboratory of Molecular Biology, Cambridge CB2 0QH, United Kingdom

## Abstract

ACBD3 is a protein localised to the Golgi apparatus and recruits other proteins, such as PI4KIIIβ, to the Golgi. However, the mechanism through which ACBD3 itself is recruited to the Golgi is poorly understood. This study demonstrates there are two mechanisms for ACBD3 recruitment to the Golgi. First, we identified that an MWT^374-376^ motif in the unique region upstream of the GOLD domain in ACBD3 is essential for Golgi localization. Second, we use unbiased proteomics to demonstrate that ACBD3 interacts with SCFD1, a Sec1/Munc-18 (SM) protein, and a SNARE protein, SEC22B. CRISPR-KO of SCFD1 causes ACBD3 to become cytosolic. We also found that ACBD3 is redundantly recruited to the Golgi apparatus by two golgins: golgin-45 and giantin, which bind to ACBD3 through interaction with the MWT^374-376^ motif. Taken together, our results suggest that ACBD3 is recruited to the Golgi in a two-step sequential process, with the SCFD1-mediated interaction occurring upstream of the interaction with the golgins.

## Introduction

The Golgi apparatus is a eukaryotic evolutionarily conserved organelle with a central role in trafficking, processing, and sorting newly synthesized proteins and lipids (Li *et al*., 2019; Liu *et al*., 2021; Park *et al*., 2021). The metazoan Golgi apparatus is a collection of Golgi stacks, with 10–20 per cell. Each Golgi stack consists of five to seven connected cisternae (Klumperman, 2011). The cisternae are polarized such that there is a *cis* and *trans* cisterna. De novo biosynthetic protein products are delivered to the *cis*-face of the Golgi apparatus and exit at the *trans*-face or from the *trans*-Golgi network (TGN). Within the Golgi apparatus, proteins are sorted into export domains for recycling back to the endoplasmic reticulum, delivery to the plasma membrane or delivery to the endolysosomal system. Accordingly, the Golgi apparatus represents a central sorting hub for protein trafficking and is essential for the sorting and delivering of many important proteins. Structural and functional changes to the Golgi apparatus are linked to numerous diseases, including developmental defects, neurodegenerative and infectious diseases, and certain cancers (Petrosyan, 2015; Liu *et al*., 2021).

Phosphoinositides are a class of phospholipids embedded in lipid bilayers in eukaryotic membranes (Hammond and Burke, 2020). Phosphatidylinositol, the precursor to all phosphoinositides, consists of two fatty acid chains attached to a glycerol backbone and an inositol head group. The inositol head group can be phosphorylated at the free hydroxyl groups at positions D3, D4, and D5 to form a large class of various phosphoinositides. These distinct phosphoinositides have a role in compartment identity and have different localizations within the endomembrane system. PI4P is crucial in the Golgi apparatus for driving several key processes through the recruitment of PI4P-interacting proteins (Santiago-Tirado and Bretscher, 2011; De Matteis *et al*., 2013; Waugh, 2019). Examples of PI4P-recruited proteins include GOLPH3 (Wood *et al*., 2009; Rahajeng *et al*., 2019) and arfaptins (Cruz-Garcia *et al*., 2013), which allow intra-Golgi trafficking and Golgi export, and clathrin adaptor proteins at the *trans*-Golgi, such as the GGA (Wang *et al*., 2007) family and AP1 (Wang *et al*., 2003). Lipid transport proteins, such as OSBP, are also partly recruited by PI4P (Levine and Munro, 2002; Mesmin *et al*., 2013; Drin, 2014; Drin *et al*., 2016). Although the most characterized role of PI4P on the Golgi apparatus is at the TGN and *trans*-Golgi, PI4P is most enriched on the *cis*-Golgi apparatus and is demonstrated to have a central role in the delivery of COPII vesicles from the ER to the *cis*-Golgi (Weixel *et al*., 2005; Lorente-Rodríguez and Barlowe, 2011).

PI4KIIIβ has the best understood role in generating PI4P at the Golgi apparatus (Waugh, 2019). PI4KIIIβ phosphorylates phosphatidylinositol at the D4 position. One of the ways that PI4KIIIβ is recruited to the Golgi apparatus is by Acyl-CoA Binding Domain Containing 3 (ACBD3, also known as GCP60 and PAP7; Sasaki *et al*., 2012). Loss of ACBD3 is lethal in mice, and ACBD3 is upregulated in multiple breast cancers in which high ACBD3 expression correlates with poor patient survival (Huang *et al*., 2018; Houghton-Gisby and J. Harvey, 2020). ACBD3 is a cytosolic protein, mainly localized to the Golgi apparatus and important for Golgi organization (Liao *et al*., 2019; Daňhelovská *et al*., 2021). ACBD3 consists of an ACBD domain, a CAR-Q domain, a unique region (UR), and a GOLD domain. ACBD3 is considered a multifunctional scaffolding protein and interacts with various proteins (Ishikawa-Sasaki *et al*., 2018; Yue *et al*., 2021), including PPM1L (Shinoda *et al*., 2012), and TBC1D22, a Rab33b GTPase activating protein (GAP) (Yue *et al*., 2017). The ACBD domain oligomerizes upon binding to C18:1-CoA or C16:0-CoA (Soupene and Kuypers, 2015) and recruits the membrane-shaping protein FAPP2 (Liao *et al*., 2019), the CAR-Q domain recruits PI4KIIIβ (Klima *et al*., 2016), and the GOLD domain and its extended UR interact with multiple different golgins including giantin, golgin-45, and golgin-160 (Sohda *et al*., 2001; Sbodio *et al*., 2006; Sbodio and Machamer, 2007; Yue *et al*., 2017). The recruitment of ACBD3 to the Golgi apparatus, therefore, represents a crucial functional step in the homeostasis and regulation of the Golgi apparatus.

There are multiple proposed mechanisms for the recruitment of ACBD3 to the Golgi apparatus. Initially, based on a yeast two-hybrid interaction, the golgin giantin was proposed to recruit ACBD3 (Sohda *et al*., 2001); however, the knockdown of giantin does not result in the loss of ACBD3 recruitment to the Golgi apparatus (Yue *et al*., 2017). Recently, the loss of ARL5B was demonstrated to cause the loss of the Golgi pool of ACBD3 (Houghton *et al*., 2022); however, ARL5B is *trans*-Golgi localized compared with the *cis*- and *trans*-Golgi localized ACBD3. Therefore, the mechanism by which ACBD3 is recruited to the Golgi apparatus remains unclear.

Here, we show that ACBD3 is recruited to the Golgi apparatus by an MWT^374-376^ motif in the “UR” upstream of the GOLD domain. We identify a host of new Golgi localized ACBD3 interactors using unbiased proteomics. We demonstrate that the SNARE SEC22B and associated SM protein SCFD1 interact with ACBD3 and abrogation of SCFD1 causes loss of ACBD3 from the Golgi apparatus. We also identify that the golgins giantin and golgin-45 interact with ACBD3 and this interaction is dependent on the MWT^374-376^ motif. Loss of both golgins results in loss of ACBD3 localization, indicating a redundant function. Our data support a novel two-step mechanism for ACBD3 recruitment to the *cis*-Golgi apparatus.

## Results

### ACBD3 is recruited to the Golgi apparatus via a protein–protein interaction within a UR of the GOLD domain

**Figure 1:**
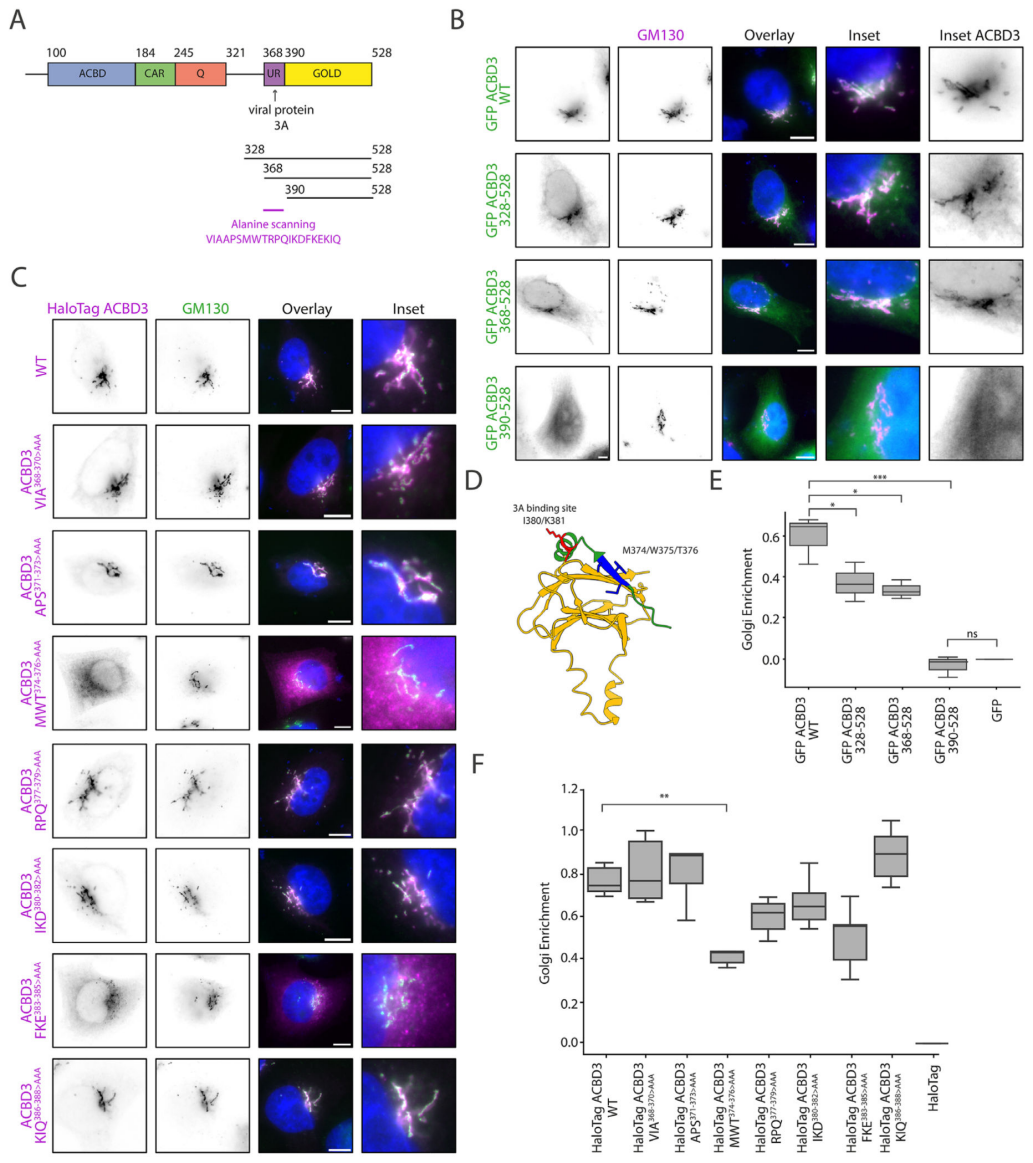
The UR of ACBD3 is essential to target ACBD3 to the Golgi apparatus. (A) Schematic of the domain organization of ACBD3. From N- to C-terminus, ACBD3 has a acyl-CoA binding domain (ACBD), a charged amino acid region (CAR) domain, a glutamine-rich domain (Q-domain), a UR, and a Golgi dynamic domain (GOLD domain). To investigate the importance of the UR for the Golgi localization of ACBD3, we generated different truncations of the UR-GOLD domain of ACBD3 and the subsequent alanine scanning approach in the UR is indicated (B and C). The UR is essential to target ACBD3 to the Golgi apparatus. Widefield imaging of HeLa cells expressing different truncations of GFP-ACBD3 (B) or different HaloTag-ACBD3 constructs mutated in the UR (alanine scanning mutagenesis) (C). The Golgi was stained with anti-GM130 antibody. Nucleus stain = DAPI. Scale bar: 10 μm. (D) Structure of the GOLD domain (yellow) and the UR (green) with MWT (M374/W375/T376) residues highlighted in blue and protein 3A targeted residues (McPhail *et al*., 2017) in red (I380/K381; AlphaFold2). (E and F) Quantitative analysis with a CellInsight CX7 high-content microscope of the Golgi localization of the different truncations and mutants of ACBD3 (B and C). The enrichment at the Golgi apparatus is calculated as defined in the methods. The experiments were performed three to five times independently. Tukey’s multiple comparisons test (HSD, FWER = 0.05) was performed. *P ≤ 0.05; **P ≤ 0.01; ***P ≤ 0.001; ns: not significant.

ACBD3 is a 528 amino acid protein containing ACBD, CAR-Q, and GOLD domains (Figure 1A) that has been reported to localize to the Golgi apparatus (Sohda *et al*., 2001; Liao *et al*., 2019; Daňhelovská *et al*., 2021; Motani *et al*., 2022). We performed high-resolution Airyscan imaging of HeLa cells immunolabeled for ACBD3 and *cis* and *trans*-Golgi markers. Despite having no transmembrane domain or other known Golgi interacting domain (Sohda *et al*., 2001), ACBD3 showed significant colocalization with *cis* and *trans*-Golgi markers (Supplemental Figure S1, A and B), consistent with previous findings (Houghton *et al*., 2022; Motani *et al*., 2022).

To identify the domain by which ACBD3 is recruited to the Golgi apparatus, we examined other known ACBD3 recruitment factors. Picornavirus 3A peptide recruits ACBD3 to viral replication sites through a protein–protein interaction via the UR of the GOLD domain of ACBD3 (Greninger *et al*., 2012; Sasaki *et al*., 2012; Klima *et al*., 2017; Lei *et al*., 2017; McPhail *et al*., 2017; Chalupska *et al*., 2019; Horova *et al*., 2019; Lyoo *et al*., 2019; Smola *et al*., 2020; Figure 1D). We reasoned that the 3A peptide must outcompete the endogenous Golgi-localized ACBD3 recruitment factor. To test this hypothesis, we generated truncations of ACBD3 in this region (Figure 1A). GFP-fused truncations that included the GOLD domain and the UR (328-528, and 368-528) are still localized to the Golgi apparatus (Figure 1B). However, the isolated GOLD domain, without the UR, is mainly cytosolic. To confirm our findings quantitatively, we built an unbiased high-throughput imaging pipeline to assess Golgi localization (Figure 1E). The different ACBD3 GFP-fused truncations were assessed for colocalization with Golgi marker GM130. Our quantitative approach confirms our observation that the UR upstream of the GOLD domain is essential for the Golgi localization of ACBD3.

We performed alanine scanning mutagenesis of the 21 amino acids of the UR (Figure 1, A, C, and D) and assessed their Golgi localization as before (Figure 1, C and F). Most of the mutants demonstrated a localization comparable to WT ACBD3 aside from one mutant, MWT^374-376>AAA^ (Figure 1D), which was relocalized to the cytosol. We thus demonstrated that the residues MWT^374-376^ in the UR of the GOLD domain of ACBD3 participate in a protein–protein interaction that recruits ACBD3 to the Golgi apparatus.

### ACBD3 interacts with an array of Golgi-localized proteins and knockout of SCFD1 causes ACBD3 to be mislocalized to the cytosol

To identify the factors responsible for the recruitment of ACBD3 to the Golgi apparatus, we performed immunoisolation of GFP-ACBD3, or the negative control GFP, using GFP-nanobody–conjugated agarose beads and assessed using mass spectrometry.

**Figure 2:**
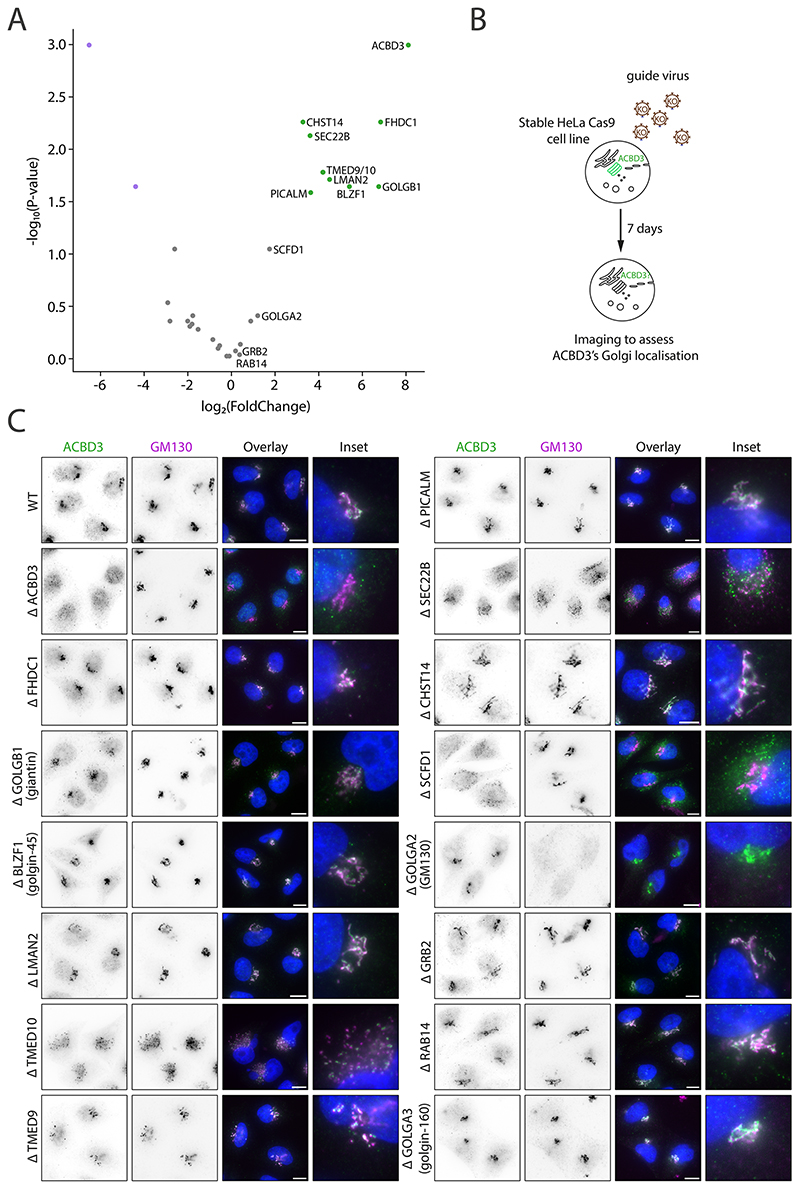
Identification of new interactors of ACBD3. (A) Volcano plot illustrating Golgi proteins identified as potential binding partners of ACBD3. Three independent GFP-trap immunoprecipitation experiments were performed on HEK-293T cells overexpressing GFP-ACBD3 or GFP alone as a control. The resultant immunoprecipitates were analyzed by mass spectrometry, and Golgi-associated proteins were identified. (B) Schematic representation of the follow-up transient CRISPR/Cas9 KO screen with the 13 first hits of proteins identified by mass spectrometry. Golgin-160 (GOLGA3), an interactor of ACBD3, was included in the screen. HeLa cells were infected with the corresponding guides and imaged 7 days postinfection. (C) Widefield imaging of endogenous ACBD3 in the different HeLa KO cells. Endogenous GM130 is stained to verify the Golgi integrity. Two biological repeats, nucleus stain = DAPI. Scale bars: 10 μm.

By definition, the unknown protein responsible for ACBD3 recruitment to the Golgi apparatus localizes to the Golgi apparatus. Therefore, interactors were bioinformatically filtered for Golgi localization using publicly available datasets (Figure 2A). Identified putative interactors included previously identified ACBD3 interactors giantin (GOLGB1) and golgin-45 (BLZF1).

To determine whether these putative interactors were necessary for the ACBD3 recruitment to the Golgi apparatus, “hits” were individually KO using CRISPR-Cas9 in HeLa cells (Figure 2, B and C). We also included golgin-160 (GOLGA3) which is a known interactor of ACBD3 (Sbodio *et al*., 2006; Sbodio and Machamer, 2007). The depletion of target proteins was verified by qPCR analysis (Supplemental Figure S2A). As previously shown, in WT cells, ACBD3 colocalized with GM130 (Figure 2C). In the positive control, CRISPR-Cas9 guides targeting ACBD3 caused loss of ACBD3 staining at the Golgi apparatus (Figure 2C; quantified in Supplemental Figure S2B). Most of the KOs showed no observable effect, and the loss of giantin did not affect the recruitment of ACBD3 to the Golgi. Loss of TMED10 and SEC22B caused a drastic loss of Golgi organization resulting in Golgi fragmentation. Loss of SCFD1, however, resulted in the almost complete loss of ACBD3 from the Golgi apparatus (Figure 2C; quantified in Supplemental Figure S2B). Therefore, we conclude that SCFD1 is a previously unidentified essential factor for the recruitment of ACBD3 to the Golgi apparatus.

### SCFD1 and ACBD3 interact upstream of PI4KIIIβ recruitment to the Golgi apparatus

**Figure 3:**
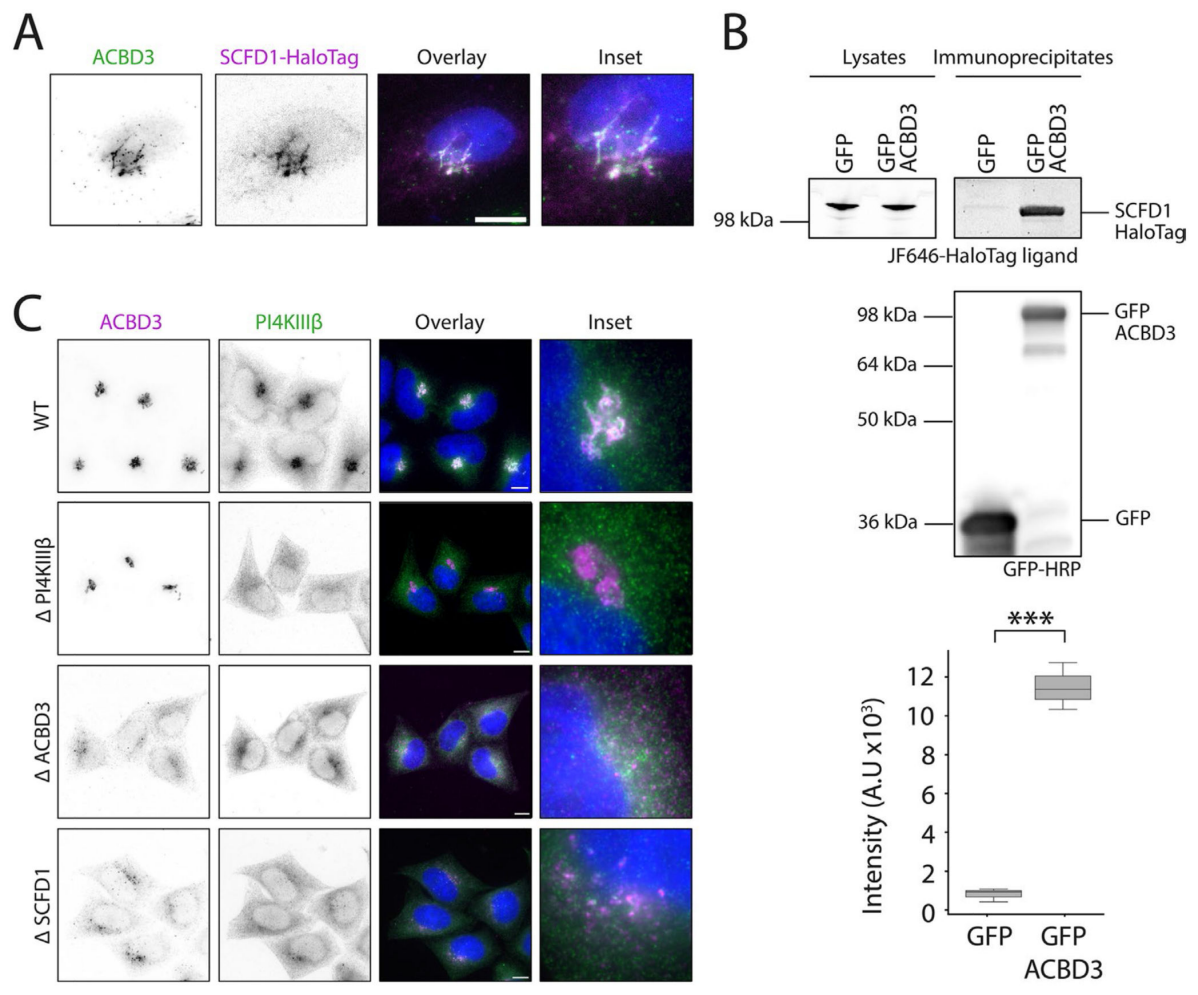
ACBD3 interacts and colocalizes with SCFD1. (A) SCFD1 colocalizes with ACBD3 at the Golgi. Widefield imaging of HeLa cells transfected with SCFD1-HaloTag and stained for endogenous ACBD3. Three biological repeats, nucleus stain = DAPI. Scale bar: 10 μm. (B) ACBD3 interacts with SCFD1. GFP alone or GFP-ACBD3 was immunoprecipitated with GFP-nanobody conjugated agarose beads from HEK-293T cells coexpressing SCFD1-HaloTag. Bound SCFD1-HaloTag and the amount of SCFD1-HaloTag in 0.125% of the lysates were detected by direct imaging of the in-gel JF646 HaloTag ligand fluorescence. The amount of GFP and GFP-ACBD3 in the immunoprecipitates was detected with an anti-GFP HRP conjugate antibody. The experiment was repeated three times, and the intensity of the bands was quantified using Fiji. The mean and SD of three independent experiments are shown and a two-tailed *t* test was performed on the data. ***P ≤ 0.001. (C) SCFD1 is important for the recruitment of PI4KIIIβ to the Golgi apparatus. Widefield imaging of endogenous ACBD3 and PI4KIIIβ in CRISPR-Cas9 KO cells of PI4KIIIβ, ACBD3, and SCFD1. Three biological repeats, nucleus stain = DAPI. Scale bars: 10 μm.

SCFD1 (aka SLY1) is a soluble cytosolic Sec1/Munc18-like protein recruited to the *cis*-Golgi apparatus to participate in catalyzing the formation of a SNARE complex (Linders *et al*., 2019; Duan *et al*., 2020a, 2020b). Exogenous overexpression of an SCFD1-HaloTag fusion colocalized with endogenous ACBD3 at the *cis*-Golgi apparatus (Figure 3A). Coexpression of SCFD1-HaloTag with GFP-ACBD3 or the negative control, GFP, and subsequent immunoprecipitation with GFP-nanobody–conjugated agarose beads corroborated mass spectrometry data that SCFD1 interacts with ACBD3 (Figure 3B).

ACBD3 recruits and activates PI4KIIIβ at the Golgi apparatus (Sasaki *et al*., 2012; Klima *et al*., 2016). Using immunostaining against endogenous proteins, we confirmed that ACBD3 colocalizes with PI4KIIIβ (Figure 3C). CRISPR-KO of PI4KIIIβ did not affect the localization of ACBD3 (Figure 3C). In agreement with previous literature, the abrogation of ACBD3 caused a loss of localization of PI4KIIIβ to the Golgi apparatus (Sasaki *et al*., 2012; Figures 3C; Supplemental Figure S2D). CRISPR-KO of SCFD1 affects both ACBD3 and PI4KIIIβ recruitment to the Golgi apparatus, with quantification revealing that ~70% of the cells have a partial or total loss of PI4KIIIβ at the Golgi. Thus, SCFD1 is upstream of ACBD3, which is upstream of PI4KIIIβ.

It has been proposed that SCFD1 interacts with the *cis*-Golgi SNARE STX5 which promotes the fusion of the incoming COPII vesicles by forming a SNARE complex with the v-SNARE SEC22B (Duan *et al*., 2020a, 2020b). Interestingly, we also identified SEC22B as a binding partner of ACBD3 in our mass spectrometry experiment (Figure 2A).

### The GOLD domain of ACBD3 interacts with the longin domain of SEC22B

**Figure 4:**
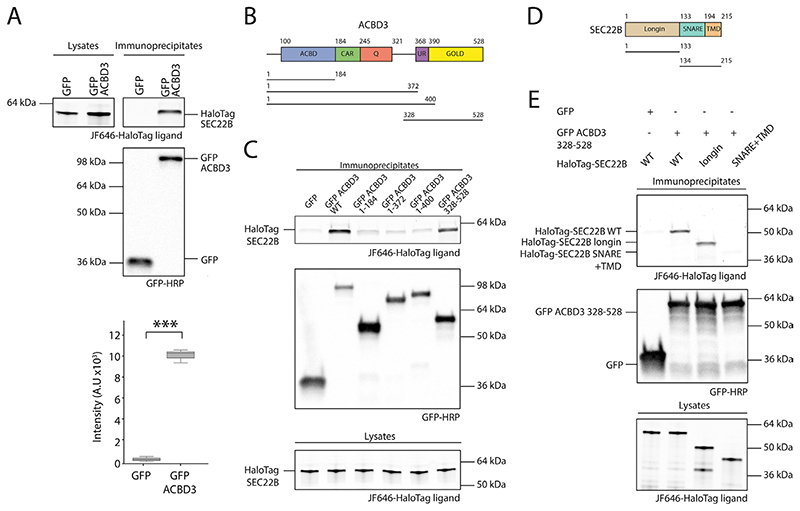
The GOLD domain of ACBD3 interacts with the longin domain of SEC22B. (A) ACBD3 interacts specifically with SEC22B. GFP alone or GFP-ACBD3 was immunoprecipitated with GFP-nanobody–conjugated agarose beads from HEK-293T cells coexpressing HaloTag-SEC22B. Bound HaloTag-SEC22B and the amount of HaloTag-SEC22B in 0.125% of the lysates were detected by directly imaging the JF646 HaloTag ligand fluorescence in-gel. The amount of GFP and GFP-ACBD3 in the immunoprecipitates was detected with an anti-GFP HRP–conjugated antibody. The experiment was repeated three times and the intensity of the bands was quantified using Fiji. The mean and SD of three independent experiments are shown and a two-tailed *t* test was performed on the data *** *P* ≤ 0.001. (B and D) Schematic of the different truncations of ACBD3 and SEC22B. (C) The GOLD domain of ACBD3 interacts with SEC22B. Different GFP-ACBD3 truncations were coexpressed with HaloTag-SEC22B in HEK-293T cells and immunoprecipitation experiments were performed as described in (A). Two biological repeats. (E) The GOLD domain of ACBD3 interacts with the longin domain of SEC22B. GFP and GFP-ACBD3 328-528 were coexpressed with different truncations of HaloTag-SEC22B in HEK-293T cells and immunoprecipitation experiments were performed as described in (A). Two biological repeats.

To confirm the interaction of ACBD3 with SEC22B, we performed coimmunoprecipitation experiments in cells expressing GFP or GFP-ACBD3 in combination with HaloTag-SEC22B (Figure 4A). GFP immunoisolation validated our mass spectrometry data that ACBD3 interacts with SEC22B. To identify which domain of ACBD3 interacts with SEC22B, we made a series of truncations to GFP-ACBD3 and performed coimmunoprecipitation experiments (Figure 4, B and C). We have, therefore, identified that ACBD3 interacts with SEC22B by the UR and GOLD domain (328-528).

SEC22B contains transmembrane, SNARE, and longin domains (Figure 4D). The transmembrane domain is buried in the lipid bilayer precluding interaction with ACBD3. To know whether ACBD3 interacts with the SNARE or longin domains, HaloTag-SEC22B truncations were generated, and a similar GFP-nanobody immunoisolation was performed as above (Figure 4E). As shown above, full-length SEC22B interacts with GFP-ACBD3 (328-528). The SNARE and transmembrane domain did not interact with ACBD3 (328-528); however, the longin domain alone does interact with ACBD3 (328-528). We thus conclude that the UR and GOLD domain of ACBD3 interacts with the longin domain of SEC22B and that ACBD3 is part of the SNARE complex involving the SM protein SCFD1 and the v-SNARE SEC22B.

### The UR in ACBD3 does not mediate the interaction to SCFD1 or SEC22B

**Figure 5:**
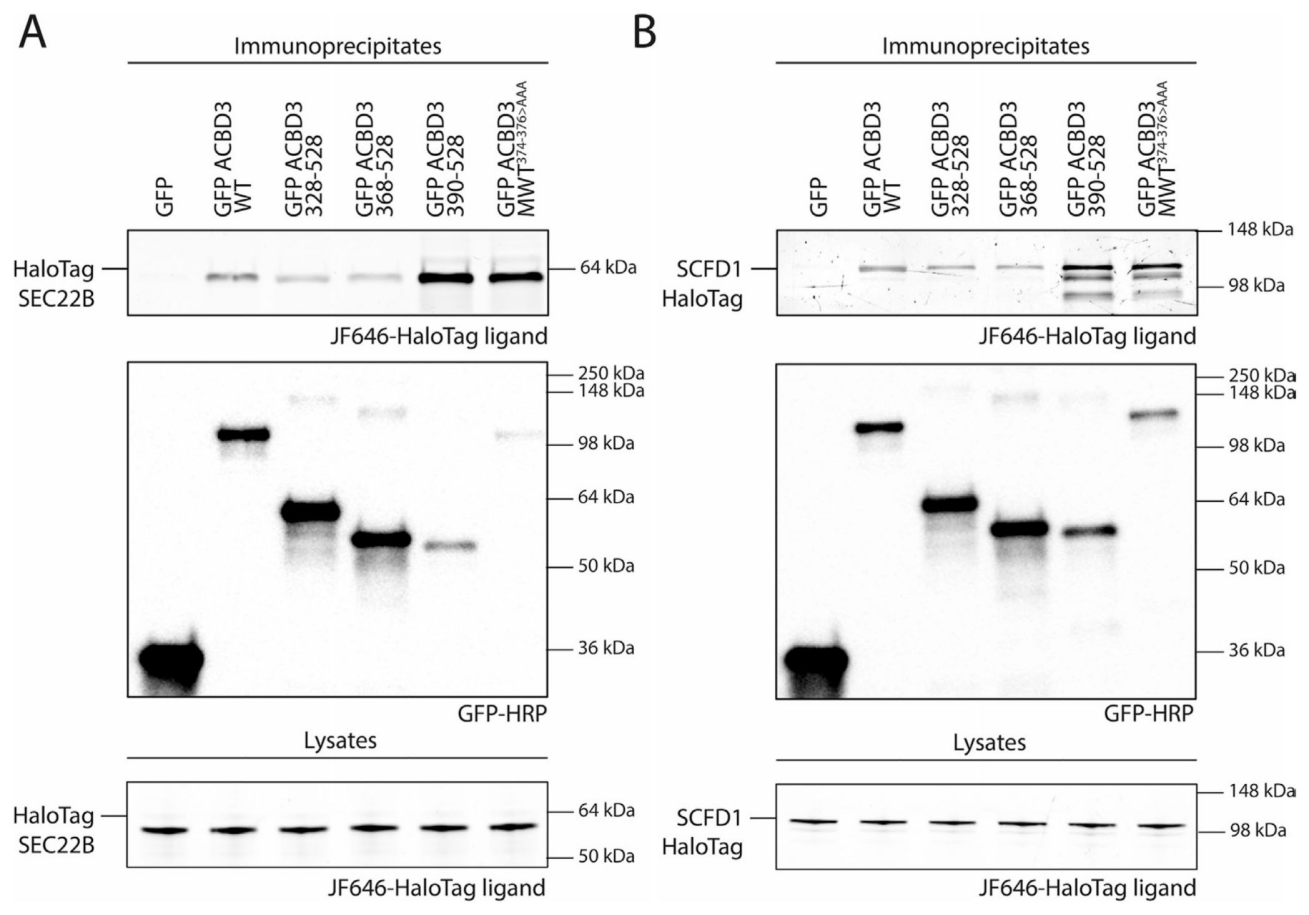
SEC22B and SCFD1 do not interact with MWT^374-376^ residues in the UR. (A and B) Different truncations of GFP-ACBD3-UR-GOLD domain and GFP-ACBD3 MWT^374-376>AAA^ mutant were co-expressed either with HaloTag-SEC22B (A) or SCFD1-HaloTag (B) in HEK-293T cells and immunoprecipitation experiments were performed as described above. Two biological repeats.

These results raised the possibility that the MWT^374-376^ residues in the UR of ACBD3 interact with SCFD1 and SEC22B to recruit ACBD3 to the Golgi apparatus. To address this, we repeated the GFP-nano-body immunoprecipitation with the series of truncations in the UR-GOLD domain of ACBD3 used previously (Figure 1A). We also included the ACBD3 MWT^374-376>AAA^ mutant, which results in the mislocalization of ACBD3 (Figure 5, A and B). Surprisingly, the cytosolic truncation of GFP-ACBD3 that does not contain the UR (390-528), and the MWT^374-376>AAA^ mutant, have a stronger interaction with both SCFD1 and SEC22B when compared with WT ACBD3. Therefore, there must be a second mechanism through which ACBD3 is recruited to the Golgi apparatus.

### Golgin-45 and giantin redundantly recruit ACBD3 to the Golgi apparatus

To understand the additional mechanism by which ACBD3 is recruited to the Golgi apparatus, we reassessed the Golgi interactome of ACBD3. Giantin (GOLGB1) has been previously demonstrated to interact with ACBD3, and we also identified it in our interactome. We also observed an interaction with golgin-45 (BLZF1), which has also been previously documented (Yue *et al*., 2017). Finally, other reports show that golgin-160 interacts with ACBD3 (Sbodio *et al*., 2006; Sbodio and Machamer, 2007), although we did not observe this in our dataset. As golgins have been documented to act redundantly (Gillingham and Munro, 2016), we hypothesized that, in addition to SCFD1, ACBD3 was being recruited to the Golgi apparatus by a combination of golgins.

**Figure 6:**
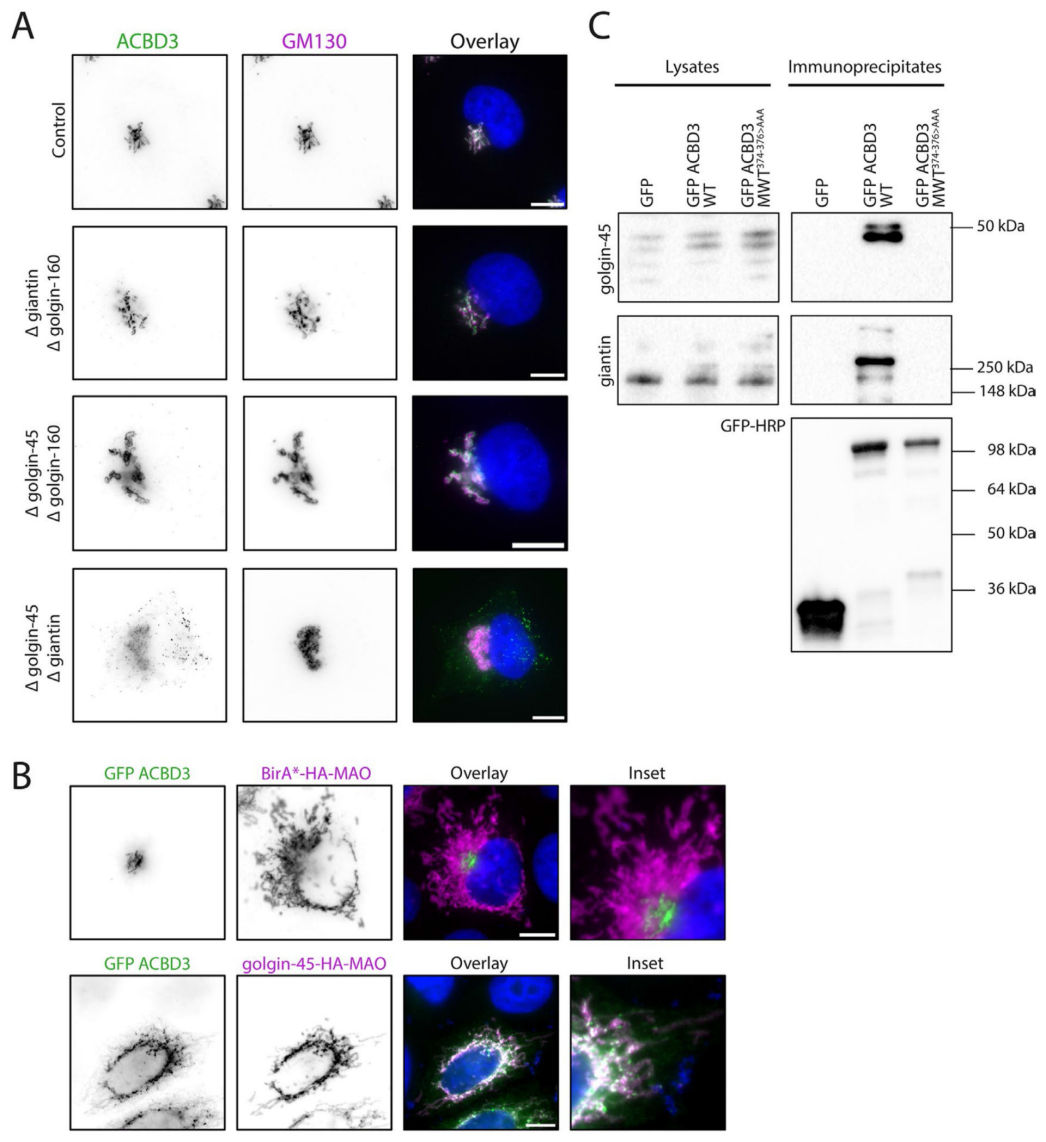
Golgin-45 and giantin interact with ACBD3 through MWT^374-376^ residues and are essential for its Golgi localization. (A) The double KO of golgin-45 and giantin affects ACBD3 localization. Widefield imaging of endogenous ACBD3 and GM130 in CRISPR-Cas9 KO cells of golgins. Two biological repeats. Scale bar: 10 μm, nucleus stain = DAPI. (B) Golgin-45 is sufficient to recruit ACBD3 to the mitochondria. Widefield imaging of mitotrap assay with coexpression of GFP-ACBD3 and golgin-45-HA-MAO. BirA*-HA-MAO was used as a negative control. Scale bar: 10 μm. Four biological repeats, nucleus stain = DAPI. (C) MWT^374-376^ residues are essential for the interaction of ACBD3 with golgins. GFP alone, GFP-ACBD3 or GFP-ACBD3 MWT^374-376>AAA^ were overexpressed in HEK-293T cells. Immunoprecipitation experiments were performed (three biological repeats) and immunoprecipitates were blotted for endogenous giantin and golgin-45.

To test whether a combination of golgins could be redundantly recruiting ACBD3 to the Golgi apparatus, we used CRISPR-Cas9 to KO combinations of the three golgins (Figure 6A). Double KOs of giantin and golgin-160, or golgin-45 with golgin-160, did not cause a loss of ACBD3 at the Golgi. However, KO of both golgin-45 and giantin drastically affects the localization of endogenous ACBD3 at the Golgi apparatus (Figure 6A; quantified in Supplemental Figure S2, B and C). This indicates that the second recruitment mechanism of ACBD3 to the Golgi apparatus is via two redundant golgins: golgin-45 and giantin.

To investigate whether there is true functional redundancy between both golgins we sought to test whether golgin-45 alone is sufficient for the recruitment of ACBD3 to intracellular organelles. To do so, we used the mitotrap assay in which golgins are ectopically localized to the mitochondrial outer membrane, where they can still capture vesicles (Wong and Munro, 2014; Figure 6B). The negative control, an exogenous enzyme BirA* targeted to the mitochondria, did not redistribute GFP-ACBD3 to the mitochondria. Ectopic expression of golgin-45 localized to the mitochondria caused a subset of the GFP-ACBD3 to be relocalized to the mitochondria from the Golgi apparatus. However, ectopic expression of golgin-45 does not seem to be sufficient to localize SCFD1-HaloTag or HaloTag-SEC22B to the mitochondria (Supplemental Figure S3, A and B). This data indicates that golgin-45 alone is sufficient to recruit a subset of ACBD3 to intracellular membranes.

We thus considered that ACBD3 MWT^374-376^ could be important for interactions with golgin-45 and giantin. We performed GFP-nanobody immunoprecipitation with a negative control (GFP), GFP-ACBD3 and GFP-ACBD3-MWT^374-376>AAA^ (Figure 6C). Immunoprecipitates were blotted for endogenous giantin and golgin-45. No interaction was observed with GFP alone, and GFP-ACBD3 interacted with both golgin-45 and giantin. Interestingly, the MWT^374-376>AAA^ mutation that prevents the localization of ACBD3 to the Golgi apparatus does not interact with either of the golgins. We therefore concluded that the second mechanism for Golgi recruitment of ACBD3 is between the MWT^374-376^ residues of ACBD3 and two golgins: golgin-45 and giantin.

### There are two sequential mechanisms for ACBD3 recruitment to the Golgi apparatus

**Figure 7:**
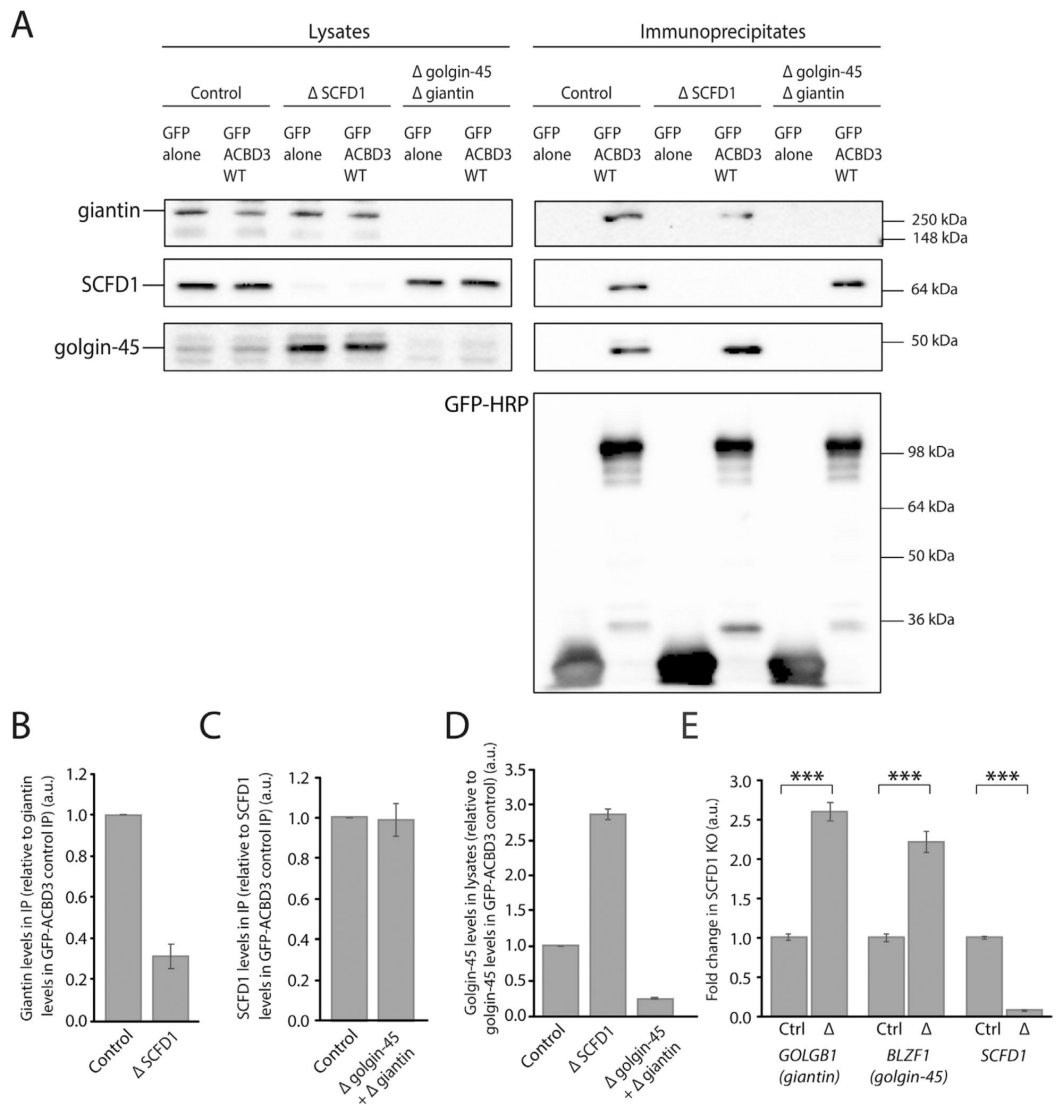
The loss of SCFD1 affects the interaction of ACBD3 with giantin. (A) GFP alone and GFP-ACBD3 were overexpressed in either WT HeLa, SCFD1-KO or a double-KO for golgin-45 and giantin. Immunoprecipitation experiments were performed, and immunoprecipitates were blotted for endogenous SCFD1, giantin, and golgin-45. The experiment was performed two times independently. (B) Quantification of giantin levels in immunoprecipitation experiment, expressed in relation to giantin levels from control condition using GFP-ACBD3 WT. An average was taken from two experiments. Error bars = SEM. (C) Same as (B) but for SCFD1. (D) Same as (B and C), but for golgin-45 levels in lysates. (E) Validation of guide RNA KO efficiency by qRT-PCR. Data from all conditions were internally normalized to GAPDH and TBP expression and are represented as fold change of control HeLa Cas9 cells. Error bar = SD of three technical repeats. ***P ≤ 0.001.

**Figure 8:**
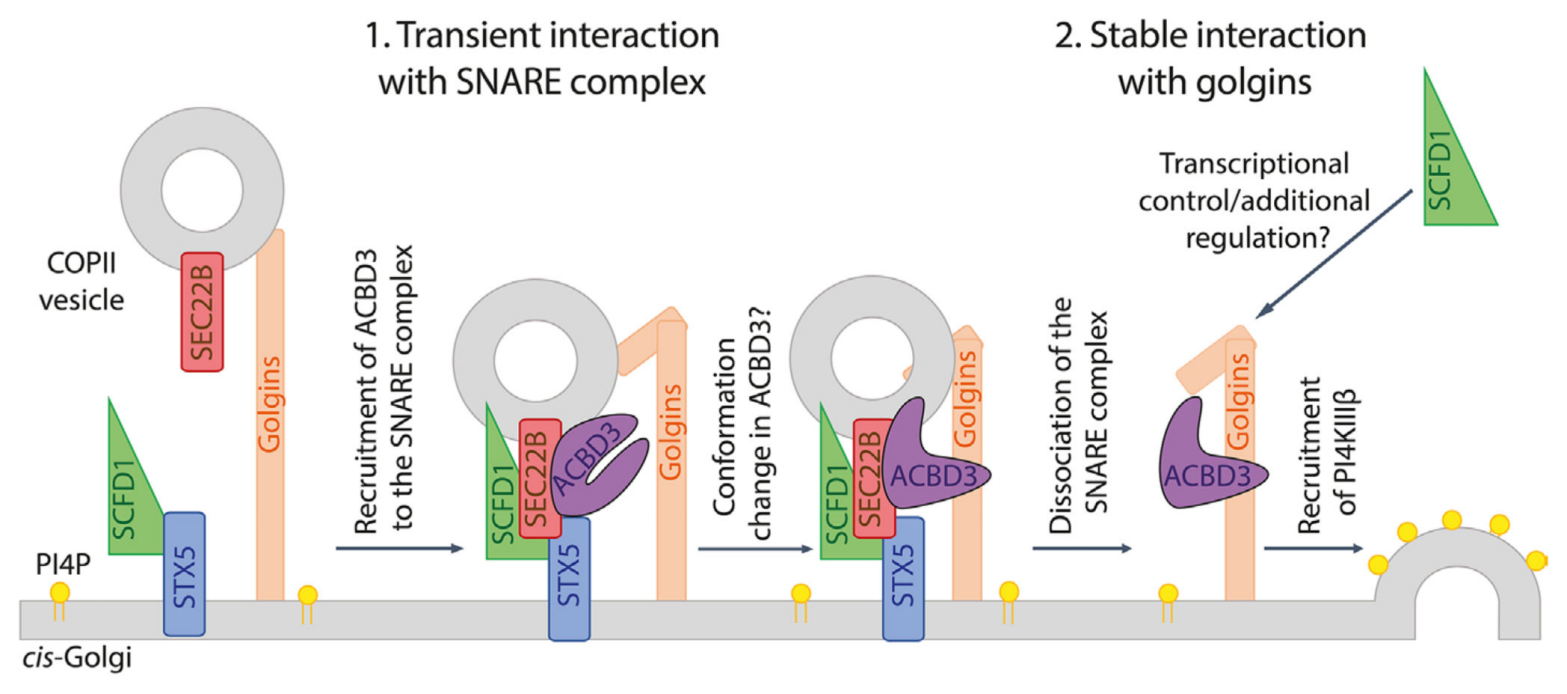
Model: ACBD3 recruitment to the *cis*-Golgi is achieved through two independent, sequential mechanisms.

We have demonstrated two mechanisms by which ACBD3 is recruited to the Golgi apparatus. The first is by SEC22B and SCFD1, which participate in a SNARE complex, and the second is by a redundant set of golgins. To experimentally interrogate whether these mechanisms are redundant or sequential, we tested GFP-ACBD3 binding to the golgins in the context of SCFD1-KO or double KO of golgin-45 and giantin (Figure 7A). The interaction between ACBD3 and giantin was decreased in the context of an SCFD1 KO (Figure 7B), whereas the interaction between ACBD3 and SCFD1 was unaffected in the context of a double golgin KO (Figure 7C). Interestingly, Golgin-45 levels were upregulated in the SCFD1 KO (Figure 7D). This was also reflected at the transcriptional level where both golgins were upregulated upon SCFD1 depletion (Figure 7E). This suggests that the recruitment of ACBD3 via SCFD1 is upstream of the interaction with the golgins, and ACBD3 is recruited to the Golgi in a two-step process. There are likely to be multiple regulatory mechanisms at play in modulating this process (Figure 8).

## Discussion

ACBD3 is a key regulator of the metazoan Golgi apparatus, yet the mechanism of its recruitment to the Golgi was unclear. Using a combination of biochemistry and genetics, we have identified two mechanisms by which ACBD3 is recruited to the Golgi apparatus. The first mechanism is through the SM protein SCFD1, which participates in a SNARE complex with SEC22B. SCFD1 and SEC22B interact with ACBD3 through the GOLD domain on the C-terminus of ACBD3. We have also identified a pair of redundant golgins: giantin and golgin-45, that are important for recruiting ACBD3. We mapped the interaction to a tripeptide MWT^374-376^ sequence in the UR of the GOLD domain of ACBD3. We demonstrate that the interaction with the golgins also depends on the presence of SCFD1, placing the SCFD1–ACBD3 interaction upstream of the golgin–ACBD3 interactions. This demonstrates a tightly regulated process allowing ACBD3 to be recruited to the Golgi with high spatiotemporal coordination.

Combining our data, we postulate a new model for the recruitment of ACBD3 to the Golgi apparatus (Figure 8). Before the fusion of a vesicle with the Golgi apparatus, the SNARE-SM complex includes at least four SNARES and the SM protein SCFD1. The presence of this SNARE-SM complex recruits ACBD3 to the localization of the incoming vesicle at the *cis*-Golgi. We propose that this “activates” ACBD3, perhaps by mediating a conformational change that exposes the MWT^374-376^ motif in the UR. Indeed, ACBD3 has been demonstrated to have multiple different conformations in solution (McPhail *et al*., 2017; Chalupska *et al*., 2019). The activated ACBD3 can thus interact with the golgins, retaining the ACBD3 at the *cis*-Golgi beyond the time of the transient *trans*-SNARE complex. The residency time of ACBD3 at the Golgi will therefore be dictated by the affinity to the golgins. Upon recruitment to the Golgi, ACBD3 will corecruit its effectors, including PI4KIIIβ. Interestingly, the presence of PI4P, the downstream product of PI4KIIIβ, is necessary for the efficient fusion of COPII vesicles at the *cis*-Golgi apparatus (Lorente-Rodríguez and Barlowe, 2011). Thus, our model suggests that ACBD3 recruits machinery at the correct place to appropriately modify the incoming vesicle membrane as it arrives in the Golgi apparatus.

One potential caveat with our model is our observation that golgin-45 can recruit ACBD3 to the mitochondrial membrane. However, this pool of ACBD3 will include both conformations. Our experiment only shows a proportion of total ACBD3 recruited to the mitochondria. Due to this, we propose this mitochondrial-recruited ACBD3 represents only a fraction of the ACBD3 pool that is in a conformation suitable for golgin-45 interaction. We also do not rule out that our model may be an oversimplification, and there may be more complicated signaling mechanisms or additional proteins that regulate the process of ACBD3 recruitment. This may be reflected by the transcriptional upregulation of both golgin-45 and giantin that is observed in SCFD1 KO cells (Figure 7E). We speculate that this may be a compensatory mechanism due to loss of SCFD1. Alternatively, this may also hint at an intriguing additional level of regulation that could play an important role in ACBD3 recruitment to the Golgi. Additionally, SCFD1 could be stabilizing the interaction between ACBD3 and the golgins, rather than acting as an independent recruitment mechanism. To validate any model as the minimum recruitment system for ACBD3 will require a set of in vitro reconstitutions that include all components in future studies.

There have been multiple other models for ACBD3 recruitment to the Golgi apparatus. Based on interaction data, ACBD3 was proposed to be recruited by giantin (Sohda *et al*., 2001). Our data and others demonstrate that although we can recapitulate an interaction with giantin, it is not solely responsible for the recruitment of ACBD3 to the Golgi apparatus. ACBD3 has also been proposed to be recruited to the Golgi apparatus by ARL5B (Houghton *et al*., 2022). Loss of ARL5B caused 50% of ACBD3 Golgi localization to be lost. The authors also demonstrated an interaction between ACBD3 and ARL5B using proximity proteomics. However, immunoprecipitation experiments between ARL5B and ACBD3 did not demonstrate an interaction. Therefore, the role of ARL5B in the recruitment of ACBD3 to the Golgi apparatus seems to be particularly to the *trans*-Golgi, where ARL5B localizes. The direct relationship between ARL5B and ACBD3 in the Golgi apparatus remains uncertain; we don’t know whether ARL5B recruits ACBD3 to the Golgi directly or whether it is upstream. Nonetheless, exploring how this interaction connects with the mechanisms discussed here might reveal an intriguing additional level of control.

One factor we have not considered in our experimental approach or model is the oligomerization of ACBD3. ACBD3 has been demonstrated to oligomerize in the presence of C18:1-CoA or C16:0-CoA (Soupene and Kuypers, 2015). Our data suggest that the binding of ACBD3 to C18:1-CoA, C16:0-CoA or related fatty acyl chains and the subsequent oligomerization does not affect its recruitment to the Golgi as deletion of the ACBD domain does not prevent Golgi localization. It is possible that this oligomerization regulates the conformational change of ACBD3, its effector recruitment, or represents an additional functional role of ACBD3, which could be explored in future studies.

Together, this study showed a highly spatiotemporally regulated mechanism for the recruitment of ACBD3 to the Golgi apparatus. We discovered previously unidentified interactors and a two-step mechanism of recruitment. Many of the identified putative novel ACBD3 interactors were not important for its recruitment to the Golgi and may represent uncharacterized ACBD3 effectors, which could be investigated in future studies. We also identified a novel MWT^374-376^ motif important for interaction with golgins; further work could investigate whether this is specific to ACBD3 or represent a common shared mechanism for protein recruitment to the Golgi.

## Materials and Methods

### Antibodies and Other Reagents

The following primary antibodies were used for IF: rabbit anti-ACBD3 (ab134952, 1:200), sheep anti-TGN46 (Bio-Rad AHP500G, 1:1000), mouse anti-GM130 (BD Biosciences 610822, 1:200), mouse anti-PI4KIIIβ (BD Biosciences 611816, 1:500), rat anti-HA (Roche 11867423001, 1:300), mouse anti-EEA1 (BD Biosciences 610456, 1:500), rabbit anti-SCFD1 (ProteinTech 12569-1-AP, 1:500) Alexa Fluor dyes (1:1000) were purchased from Invitrogen (donkey anti-rabbit 488: A32790; donkey anti-sheep 647: A21448; donkey anti-mouse 488: A32766; donkey anti-mouse 647: A32787, donkey anti-rat 647: A48272).

The following primary antibodies were used for Western blot analysis in this study: mouse anti-GFP HRP–conjugated antibody (Miltenyi Biotec 130-091-833, 1:1000), rabbit anti-golgin-45 (Abcam ab155510, 1:1000), rabbit anti-giantin (Sigma HPA011008, 1:1000), rabbit anti-SCFD1 (Abcam ab86594, 1/1000). Goat HRP-conjugated secondary antibodies (1:5000) were purchased from Abcam (anti-mouse: ab205719; anti-rabbit: ab205718).

The following dyes were obtained from these vendors: Janelia Fluor HaloTag Ligands (GA112A; Promega), DAPI (D21490; Invitrogen), HCS CellMask Blue Stain (H32720; Invitrogen).

The following antibiotics were used to select cell lines: puromycin (1 μg/ml for HeLa cells and 0.5 μg/ml for HEK293T cells; A1113803; Life Technologies) and blasticidin (15 μg/ml for HeLa cells and 5 μg/ml for HEK293T cells; A1113903; Life Technologies).

### Plasmids

pHaloTag-C1 and pHaloTag-N1 were generated by replacing the eGFP in Clontech vectors with HaloTag using Gibson assembly (E2621L; New England Biolabs). HaloTag-ACBD3, SCFD1-HaloTag and HaloTag-SEC22B were cloned by Gibson assembly using a synthetic codon optimized version of the gene of interest (Integrated DNA Technologies), cloned in-frame upstream or downstream of HaloTagTag in pHaloTag-C1 or pHaloTag-N1. GFP-ACBD3 was generated with the same approach except that peGFP-C1 vector was used.

For the alanine scanning approach in the UR of ACBD3, alanine mutations were introduced into the HaloTag-ACBD3 vector by using the Q5 Site-Directed Mutagenesis Kit from New England Biolabs. The Q5 Hot Start High-Fidelity 2X Master Mix (M0494S) was used along with custom mutagenic primers to introduce substitutions of target residues in ACBD3 by PCR (Halo-ACBD3 368-370 [VIA^368-370>AAA^], 371-373 [APS^371-373>AAA^], 374-376 [MWT^374-376>AAA^], 377-379 [RPQ^377-379>AAA^], 380-382 [IKD^380-382>AAA^], 383-385 [FKE^383-385>AAA^], 386-388 [KIQ^386-388>AAA^]). The PCR product was used for reaction with the Kinase-Ligase-*Dpn*I (KLD) enzyme mix (M0554S). Truncations of GFP-ACBD3 (1-184, 1-372, 1-400, 328-528, 368-528, and 390-528) and HaloTag-SEC22B (isolated longin and SNARE and transmembrane domains) were also generated with the Q5 Site-Directed Mutagenesis Kit.

BirA*-HA-MAO, and golgin-45-HA-MAO were a kind gift from the Sean Munro lab. BirA*-HA-MAO was generated as described previously (Gillingham *et al*., 2019). Golgin-45-HA-MAO was generated by Gibson assembly using the HA-MAO vector digested with *Nhe*l/*Kpn*l and golgin-45 cDNA (missing its C-ter, amino acids 1 to 368) purchased from Origene.

Cas9 viral expression backbone was a kind gift from Paul Lehner lab as well as the packaging vectors pMD.G and pCMVR8.91.

pKLV-U6gRNA(BbsI)-PGKblast2ABFP vector was generated by replacing the puromycin-resistant gene in pKLV-U6gRNA(BbsI)-PGKpuro2ABFP (Addgene plasmid #50946) with blasticidin via Gibson assembly.

To generate the piggybac-GFP and GFP-ACBD3 constructs, a PCR product of GFP and GFP-ACBD3 were cloned into the piggybac-CMV-StrepKDEL-IRES-SBP-HaloTag construct (Pereira *et al*., 2023) digested with *Afe*l and *Xba*l. The piggybac transposon system was used to generate stable HeLa Cas9 GFP and GFP-ACBD3 cell lines. These were further selected *via* live cell flow cytometry for a similar level of expression.

Plasmids and primers used in this work are available upon request. All constructs were sequenced to verify their integrity.

### Cell lines and lentiviral particles production

All cell lines were grown in DMEM high glucose (D6429; Sigma-Aldrich) supplemented with 10% fetal bovine serum (FBS; F7424; Sigma-Aldrich) and MycoZap Plus-CL (VZA-2012; Lonza). They were kept at 37°C in a humidified 5% CO_2_ atmosphere.

HeLa cells were already available in the lab; HEK293T cells were a gift from Janet Deane (CIMR, University of Cambridge, UK); Lenti-X 293T cells were obtained from Takara Bio (632180).

Lenti-X 293T cells were used to package pKLV-U6gRNA(BbsI)-PGKpuro2ABFP or pKLV-U6gRNA(BbsI)-PGKblast2ABFP vectors encoding guide RNAs into lentiviral particles as previously described (Pirona *et al*., 2020). The IDT Alt-R CRISPR-Cas9 guide RNA tool and CRISPOR (http://crispor.tefor.net/) were used to custom design two guide sequences per gene of interest (except for GOLGB1/giantin and BLZF1/golgin-45 where only one guide sequence was used, see Supplemental Table S3). Viral supernatants were harvested after 48 h, filtered through a 0.45-μm filter, and when needed, concentrated down 10 or 30 times using the Lenti-X Concentrator (631232; Takara Bio). Supernatants were kept at –80°C before being directly applied to target cells which were then spun at 700 xg for 1 h at 37°C. When possible, cells were transiently selected with the appropriate antibiotic 48 h posttransduction.

Stable HeLa Cas9-expressing cell lines were generated by infecting cells with lentiviral particles carrying Cas9 plasmid DNA (gift from Paul Lehner) followed by selection for blasticidin antibiotic resistance. Cas9 expression on >90% of the cell population was further confirmed through flow cytometry analysis, by testing loss of cell surface expression of beta-2 microglobulin, upon transduction with lentiviral particles containing a beta-2 microglobulin targeting guide RNA, using a mouse monoclonal anti-B2M antibody (gift from Paul Lehner).

### GFP trap experiment followed by Mass Spectrometry analysis

1 × 10^7^ HEK293T cells were seeded onto a 15-cm dish (two per condition). On d 4, cells were transfected with GFP or GFP-ACBD3 by using PEI (Polyethylenimine branched, 408727, Sigma-Aldrich). The following day, plates were washed once with PBS1X and incubated with 5 ml PBS1X (w/o Ca^2+^ and MgCl^2^) for 10 min at 37°C. Cells were collected and the plates washed two times with 5 ml PBS1X. Cells from the same condition were pooled together and were pelleted at 500 × *g* for 3 min and resuspended with 450 μl of lysis buffer (10 mM Tris pH7.4, 150 mM NaCl, 1 mM MgCl2, 0.5% TX100, cOmplete EDTA-free Protease Inhibitor Cocktail (Roche, 04693132001). After 30-min incubation on ice, 600 μl of lysis buffer missing TX-100 was added and the lysates were spun for 20 min at 17 000 × *g* at 4°C. Supernatants were incubated for 1 h with 20 μl prewashed GFP-Trap Agarose beads (ProteinTech, gta-10). Beads were pelleted and washed two times with 1 ml of lysis buffer containing 0.2% TX-100 for 5 min on a turning wheel at 4°C followed by four quick washes with 1 ml of lysis buffer missing TX-100. Fifty-microliters of Laemmli buffer + dithiothreitol (DTT; Bio-Rad, 1610747) was added to the beads and samples were boiled for 10 min at 95°C. Beads were removed and the eluates were analyzed on a Q Exactive Plus Orbitrap Mass Spectrometer (Thermo Fisher Scientific). Protein hits were filtered for Golgi-associated proteins as per Uniprot annotations. This experiment was performed three times independently. See postanalysis data set before and after Golgi filter (Supplemental Tables S1 and S2, respectively).

### Immunoprecipitation experiments

4 × 10^6^ HEK293T cells were seeded per condition onto a 10-cm dish. On d 3, cells were transfected with GFP alone or GFP-ACBD3 (wild-type, truncations, and mutants) together with SCFD1-HaloTag or HaloTag-SEC22B (and isolated domains) by using PEI. Cells were incubated overnight with complete DMEM containing JF646 Halo-Tag ligand (20 nM; GA112A; Promega). The GFP protein isolation was performed as mentioned above except that only 10 μl of GFP-Trap Agarose beads were used which were blocked for 1 h with 3% bovine serum albumin (BSA) at 4°C. Beads were washed three times with 1 ml of lysis buffer containing 0.2% TX-100 (one quick wash and two washes of 5 min) followed by one quick wash with 1 ml of PBS1X. 50 μl of Laemmli buffer + DTT was added to the beads and samples were boiled for 10 min at 95°C. 1.25 μl of the lysates (0.125%) and 20–25 μl of the immunoprecipitates were resolved on a gradient Tris-Glycine acrylamide gel. The gel was directly imaged at 647 nm to detect SCFD1-HaloTag or HaloTag-SEC22B (Chemi-Doc Imaging System, Bio-Rad). The gel was then transferred to a polyvinylidene difluoride (PVDF) membrane which was blocked with 5% skimmed milk and incubated with anti-GFP HRP conjugate antibody overnight at 4°C. Membranes were washed with PBS-T in-between steps, and finally, their immunoreactivity was visualized using Clarity (1705061; Bio-Rad) or WesternBright Sirius (K-12043-D10; Advansta) ECL substrate in the ChemiDoc Imaging System (Bio-Rad). Either Fiji or Image Lab software (version 6.1, Bio-Rad) were used to quantify immunoblot data.

For the immunoprecipitation experiments investigating the interaction of ACBD3 with endogenous golgin-45 and giantin, HEK293T cells were transfected with GFP alone, GFP-ACBD3 WT and MWT^374-376>AAA^, and the experiment was carried out as described above except that 60 μl of Laemmli buffer + DTT was added to the beads. 15 μl of the lysates and 20 μl of the immunoprecipitates were resolved on a 8% Tris-Glycine acrylamide gel and transferred to a PVDF membrane. Membranes were incubated with an anti-golgin-45 or anti-giantin antibody overnight at 4°C, followed by a 1-h incubation with a secondary antibody at room temperature.

For the immunoprecipitation experiments investigating the interaction of ACBD3 with endogenous golgin-45 and giantin in SCFD1 and double golgin-45/giantin KO cell lines, guide RNAs targeting SCFD1, golgin-45 and giantin were cloned into pKLV-U6gRNA (*Bbs*l)-PGKpuro2ABFP and viral particles were produced as described above. For the SCFD1 KO condition, 2 × 10^5^ of stable HeLa Cas9 GFP or GFP-ACBD3 cells were infected with 250 μl of a 10x concentrated lentiviral supernatant in a six-well plate (in a final volume of 2 ml). For the double golgin-45/giantin KO condition, 200 × 10^3^ of stable HeLa Cas9 GFP or GFP-ACBD3 cells were infected (250 μl of a 10x and 150 μl of a 30x concentrated lentiviral supernatant for golgin-45 and giantin, respectively). Seventy-two hours posttransduction, cells of the same condition were pooled together, replated onto a 10-cm dish and selected with puromycin. On d 7, the immunoprecipitation experiment was carried out as described above for HEK293T cells, except that trypsin was used to lift the cells and the same amount of cells were used for each condition. 20 μl of the lysates and the total amount of the immunoprecipitates were resolved on a 8% Tris-Glycine acrylamide gel and transferred to a PVDF membrane. Membranes were incubated with an anti-golgin-45 or anti-giantin antibody overnight at 4°C, followed by a 1-h incubation with a secondary antibody at room temperature.

### Targeted CRISPR KO screen

To generate transient KO cells of the different genes identified by mass spectrometry, guide RNAs targeting the gene of interest were cloned into pKLV-U6gRNA (BbsI)-PGKblast2ABFP. Viral particles were produced with Lenti-X 293T cells as described above. The HeLa Cas9 stable cell line was then transduced. Briefly, 1 × 10^4^ HeLa Cas9 cells were infected with 250 μl of lentiviral supernatant in a 96-well plate. Seventy-two hours posttransduction cells were replated in a 48-well plate and incubated for an extra 2 d. On d 6, 2 × 10^4^ of transduced cells were plated onto Matrigel-coated (1:100 in complete DMEM; 354277; Corning) glass coverslips (400-03-19; Academy). On d 8, cells were fixed with 4% paraformaldehyde for 15 min. Cells were permeabilized with 0.1% TX-100 (T9284; Sigma-Aldrich) and incubated for 1 h with a blocking solution containing 3% BSA (BP9703-100; Sigma-Aldrich). Cells were incubated for 1 h with anti-ACBD3 and anti-GM130 primary antibodies followed by 1 h incubation with appropriate Alexa Fluor Dyes. PBS1X was used to wash cells in-between all steps and DAPI stained (300 nM; D21490 Invitrogen) for 5 min. Coverslips were mounted in ProLong Gold (P36930; Life Technologies).

### Quantification of ACBD3 localization at the Golgi apparatus by automated microscopy

6.7 × 10^3^ HeLa cells were plated in 96-well imaging plates (Viewplates, Perkin Elmer). The next day, cells were transfected using Fu-GENE 6 with GFP-ACBD3 WT and the different truncations (1-184, 1-372, 1-400, 328-528, 368-528, 390-528) or with HaloTag-ACBD3 WT and the different alanine mutants. 4 h post-transfection, media was replaced with fresh complete DMEM containing JF646 HaloTag ligand (20 nM; GA112A; Promega) when required. On the following day, cells were fixed with cytoskeletal fixing buffer (300 mM NaCl, 10 mM EDTA, 10 mM Glucose, 10 mM MgCl_2_, 20 mM PIPES pH 6.8, 2% Sucrose, 4% paraformaldehyde) for 15 min and processed for immunostaining as described above. Anti-GM130 primary antibody was used as well as a HCS CellMask Blue Stain (H32720; Invitrogen). Cells were stored in PBS1X at 4°C until imaged.

When using KO cells, 1 × 10^4^ HeLa Cas9 cells were infected with 25 μl (for SCFD1 and golgin-45) or 45 μl (for giantin) of 10x concentrated lentiviral supernatant in a 96-well plate. Seventy-two hours posttransduction, cells were replated onto a 48-well plate and selected with puromycin. On d 6, cells were seeded onto a 96-well Viewplate and, on d 8, immunostaining was carried out by using anti-GM130 and anti-ACBD3 primary antibodies and a HCS CellMask Blue Stain.

Images were acquired on a CellInsight CX7 high-content microscope (Thermo Fisher Scientific) using a 20 × objective (NA 0.45, Olympus). The Cell Mask was used for automated focusing and cell detection. Forty to 60 fields were imaged in three wells for each condition. Each experiment was performed three to five times independently. The general intensity measurement algorithm from the HCS Studio software was used as a template with parameters adapted to our experiments. Cells were defined by the Cell Mask stain, and the Golgi apparatus was masked using the anti-GM130 antibody labeling (with the Circ function). For exogenous ACBD3 expression, cells transfected with GFP-ACBD3 or HaloTag-ACBD3 were selected with a fixed area and with a fixed average intensity. The mean intensity of ACBD3 was measured using the previously obtained cell and Golgi masks. Raw data was exported to an Excel file, and the ratio of the average mean intensity of ACBD3 at the Golgi/the average mean intensity of total ACBD3 in the cell was calculated. The ratio of HaloTag or GFP alone (control) was then subtracted from all corresponding ratios, and a final mean ratio comprehending all biological repeats was obtained. We term this “Golgi enrichment”. To account for variation in ACBD3 levels in the KO cell samples, only the mean intensities of ACBD3 at the Golgi were considered and averaged. A minimum of three biological repeats were carried out for each experiment.

### Immunofluorescence and Microscopy

For immunofluorescence microscopy, 4 × 10^4^ HeLa cells were plated onto Matrigel-coated glass coverslips in a 24-well plate. FuGENE 6 was used to transfect constructs encoding proteins of interest. Four hours posttransfection media was replaced with fresh complete DMEM containing JF646 HaloTag ligand when required. On the following day, cells were fixed with cytoskeletal fixing buffer and immunostaining was carried out as described above.

For KO of specific genes, 1 × 10^4^ HeLa Cas9 cells were infected with 250 μl of lentiviral supernatant in a 96-well plate. Seventy-two hours posttransduction, cells were replated onto a 48-well plate and selected with puromycin. On d 6, cells were seeded onto Matrigel-coated glass coverslips in a 24-well plate and immunostaining was carried out as described above.

Standard epifluorescence images were obtained with an AxioImagerZ2 microscope (Zeiss) equipped with an Orca Flash 4.0v2 camera (Hamamatsu), an HXP 120W light source and a 100 × 1.4 NA Plan-Apochromat objective, all under the control of ZEN Blue software (Zeiss).

To analyze the colocalization of ACBD3 with different compartment markers (TGN46, GM130, and EEA1), cells were imaged on a LSM880 confocal with Airyscan (Zeiss) with a 63 × 1.4NA Plan-Apochromat objective. Images were Airyscan processed with a fixed strength parameter set at six. Colocalization analyses were performed using the Fiji software plugin Coloc 2 to determine the Pearson’s correlation coefficient among two channels from three independent experiments. The Fiji software plugin Plot profile was used to generate the fluorescence line profiles.

### RNA extraction and qPCR

Total RNA was isolated from cultured HeLa Cas9 WT or KO cells (35-mm dish), using the RNeasy Mini Kit (74104; Qiagen) according to the manufacturer’s instructions. RNA quantification was performed using NanoDrop One (Thermo Fisher Scientific). Strand cDNA was generated by priming 1 μg of total RNA with a oligo (dT)16/random hexamers mix, using the High-Capacity RNA-to-cDNA Kit (4387406; Applied Biosystems), following manufacturer’s instructions. cDNA templates were diluted 50-fold and 5 μl was used with specific oligos spanning two continuous exons (Supplemental Table S4) along with the PowerUp SYBR Green Master Mix (A25741; Applied Biosystems) for the qPCR reaction. All reactions were performed in three technical replicates using the CFX96 Touch Real-Time PCR Detection System (Bio-Rad). Data was analyzed according to the 2^-ΔΔCT^ method (Livak and Schmittgen, 2001).

### Statistical Analysis

Statistical analysis was performed using Python 3.9 and statistical significance was considered when *P* < 0.05. Comparisons were made using either Student’s *t* test or Multiple Comparison of Means - Tukey HSD, FWER = 0.05. These were conducted in Python 3.9 using pandas, matplotlib, seaborn and scipy. We used the Multi-Comparison function in scipy, and the comp.tukeyhsd for the posthoc adjustment. All quantitative data are expressed as mean ± SD of at least three independent experiments.

## Supplementary Material

Supplementary Data 1

Supplementary Data 2

Supplementary Data 3

Supplementary Data 4

Supplementary Information

## Significance Statement

- ACBD3, a Golgi-localized protein, plays a vital role in recruiting essential proteins like PI4KIIIβ. However, how ACBD3 itself gets to the Golgi was previously unclear. Our study sheds light on a two-step mechanism for ACBD3’s Golgi recruitment.
- We identified a crucial MWT motif in ACBD3 and discovered a new interacting partner, SCFD1 and SEC22B, that functions upstream of golgin-45 and giantin to recruit ACBD3.
- This two-step process not only reshapes our understanding of Golgi dynamics but also highlights the potential therapeutic avenues that might be explored in conditions where the proper functioning of the Golgi is crucial.

## Abbreviations used

ACBD: acyl-CoA binding domain
ACBD3: Acyl-CoA Binding Domain Containing 3
AP1: adaptor protein 1
CAR domain: charged amino acid region domain
GOLD domain: Golgi dynamic domain
KO: knock-out
OBSP: oxysterol-binding protein
Q domain: glutamine-rich domain
SM: Sec1/Munc-18
TGN: trans-Golgi network
UR: unique region

## References

[R1] Chalupska D, Różycki B, Klima M, Boura E (2019). Structural insights into Acyl-coenzyme A binding domain containing 3 (ACBD3) protein hijacking by picornaviruses. Protein Sci.

[R2] Cruz-Garcia D, Ortega-Bellido M, Scarpa M, Villeneuve J, Jovic M, Porzner M, Balla T, Seufferlein T, Malhotra V (2013). Recruitment of arfaptins to the trans-Golgi network by PI(4)P and their involvement in cargo export. EMBO J.

[R3] Daňhelovská T, Zdražilová L, Štufková H, Vanišová M, Volfová N, Křížová J, Kuda O, Sládková J, Tesařová M (2021). Knock-Out of ACBD3 leads to dispersed Golgi structure, but unaffected mitochondrial functions in HEK293 and HeLa Cells. Int J Mol Sci.

[R4] De Matteis MA, Wilson C, D’Angelo G (2013). Phosphatidylinositol-4-phosphate: the Golgi and beyond. Bioessays.

[R5] Drin G (2014). Topological regulation of lipid balance in cells. Annu Rev Biochem.

[R6] Drin G, Moser von Filseck J, Copic A (2016). New molecular mechanisms of inter-organelle lipid transport. Biochem Soc Trans.

[R7] Duan M, Gao G, Banfield DK, Merz AJ (2020a). Golgi SM protein Sly1 promotes productive trans-SNARE complex assembly through multiple mechanisms. bioRxiv.

[R8] Duan M, Plemel RL, Takenaka T, Lin A, Delgado BM, Nattermann U, Nickerson DP, Mima J, Miller EA, Merz AJ (2020b). Gatekeeper helix activates Golgi SM protein Sly1 and directly mediates close-range vesicle tethering. bioRxiv.

[R9] Gillingham AK, Bertram J, Begum F, Munro S (2019). In vivo identification of GTPase interactors by mitochondrial relocalization and proximity biotinylation. eLife.

[R10] Gillingham AK, Munro S (2016). Finding the Golgi: Golgin coiled-coil proteins show the way. Trends Cell Biol.

[R11] Greninger AL, Knudsen GM, Betegon M, Burlingame AL, Derisi JL (2012). The 3A protein from multiple picornaviruses utilizes the golgi adaptor protein ACBD3 to recruit PI4KIIIβ. J Virol.

[R12] Hammond GRV, Burke JE (2020). Novel roles of phosphoinositides in signaling, lipid transport, and disease. Curr Opin Cell Biol.

[R13] Horova V, Lyoo H, Różycki B, Chalupska D, Smola M, Humpolickova J, Strating J, van Kuppeveld FJM, Boura E, Klima M (2019). Convergent evolution in the mechanisms of ACBD3 recruitment to picornavirus replication sites. PLoS Pathog.

[R14] Houghton FJ, Makhoul C, Cho EH-J, Williamson NA, Gleeson PA (2022). Interacting partners of Golgi-localized small G protein Arl5b identified by a combination of in vivo proximity labelling and GFP-Trap pull down. FEBS Lett.

[R15] Houghton-Gisby J, Harvey AJ (2020). ACBD3, its cellular interactors, and its role in breast cancer. Cancer Stud Ther J.

[R16] Huang Y, Yang L, Pei Y-Y, Wang J, Wu H, Yuan J, Wang L (2018). Over-expressed ACBD3 has prognostic value in human breast cancer and promotes the self-renewal potential of breast cancer cells by activating the Wnt/beta-catenin signaling pathway. Exp Cell Res.

[R17] Ishikawa-Sasaki K, Nagashima S, Taniguchi K, Sasaki J (2018). Model of OSBP-mediated cholesterol supply to Aichi Virus RNA replication sites involving protein-protein interactions among viral proteins, ACBD3, OSBP, VAP-A/B, and SAC1. J Virol.

[R18] Klima M, Tóth DJ, Hexnerova R, Baumlova A, Chalupska D, Tykvart J, Rezabkova L, Sengupta N, Man P, Dubankova A (2016). Structural insights and in vitro reconstitution of membrane targeting and activation of human PI4KB by the ACBD3 protein. Sci Rep.

[R19] Klima M, Chalupska D, Różycki B, Humpolickova J, Rezabkova L, Silhan J, Baumlova A, Dubankova A, Boura E (2017). Kobuviral non-structural 3A proteins act as molecular harnesses to hijack the host ACBD3 protein. Structure.

[R20] Klumperman J (2011). Architecture of the mammalian Golgi. Cold Spring Harb Perspect Biol.

[R21] Lei X, Xiao X, Zhang Z, Ma Y, Qi J, Wu C, Xiao Y, Zhou Z, He B, Wang J (2017). The Golgi protein ACBD3 facilitates Enterovirus 71 replication by interacting with 3A. Sci Rep.

[R22] Levine TP, Munro S (2002). Targeting of Golgi-specific pleckstrin homology domains involves both PtdIns 4-kinase-dependent and-independent components. Curr Biol.

[R23] Liao J, Guan Y, Chen W, Shi C, Yao D, Wang F, Lam SM, Shui G, Cao X (2019). ACBD3 is required for FAPP2 transferring glucosylceramide through maintaining the Golgi integrity. J Mol Cell Biol.

[R24] Li J, Ahat E, Wang Y (2019). Golgi structure and function in health, stress, and diseases. Results Probl Cell Differ.

[R25] Linders PT, van der Horst C, Beest MT, van den Bogaart G (2019). Stx5-Mediated ER-Golgi transport in mammals and yeast. Cells.

[R26] Liu J, Huang Y, Li T, Jiang Z, Zeng L, Hu Z (2021). The role of the Golgi apparatus in disease (Review). Int J Mol Med.

[R27] Livak KJ, Schmittgen TD (2001). Analysis of relative gene expression data using real-time quantitative PCR and the 2(-Delta Delta C(T)) Method. Methods.

[R28] Lorente-Rodríguez A, Barlowe C (2011). Requirement for Golgi-localized PI(4)P in fusion of COPII vesicles with Golgi compartments. MBoC.

[R29] Lyoo H, van der Schaar HM, Dorobantu CM, Rabouw HH, Strating J, van Kuppeveld FJM (2019). ACBD3 is an essential Pan-enterovirus host factor that mediates the interaction between viral 3A protein and cellular protein PI4KB. MBio.

[R30] McPhail JA, Ottosen EH, Jenkins ML, Burke JE (2017). The molecular basis of Aichi Virus 3A protein activation of Phosphatidylinositol 4 Kinase IIIβ, PI4KB, through ACBD3. Structure.

[R31] Mesmin B, Bigay J, Moser von Filseck J, Lacas-Gervais S, Drin G, Antonny B (2013). A four-step cycle driven by PI(4)P hydrolysis directs sterol/PI(4)P exchange by the ER-Golgi tether OSBP. Cell.

[R32] Motani K, Saito-Tarashima N, Nishino K, Yamauchi S, Minakawa N, Kosako H (2022). The Golgi-resident protein ACBD3 concentrates STING at ER-Golgi contact sites to drive export from the ER. Cell Rep.

[R33] Park K, Ju S, Kim N, Park S-Y (2021). The Golgi complex: a hub of the secretory pathway. BMB Rep.

[R34] Pereira C, Stalder D, Anderson GSF, Shun-Shion AS, Houghton J, Antrobus R, Chapman MA, Fazakerley DJ, Gershlick DC (2023). The exocyst complex is an essential component of the mammalian constitutive secretory pathway. J Cell Biol.

[R35] Petrosyan A (2015). Onco-Golgi: Is fragmentation a gate to cancer progression?. Biochem Mol Biol J.

[R36] Pirona AC, Oktriani R, Boettcher M, Hoheisel JD (2020). Process for an efficient lentiviral cell transduction. Biol Methods Protoc.

[R37] Rahajeng J, Kuna RS, Makowski SL, Tran TTT, Buschman MD, Li S, Cheng N, Ng MM, Field SJ (2019). Efficient Golgi forward trafficking requires GOLPH3-driven, PI4P-dependent membrane curvature. Dev Cell.

[R38] Santiago-Tirado FH, Bretscher A (2011). Membrane-trafficking sorting hubs: cooperation between PI4P and small GTPases at the trans-Golgi network. Trends Cell Biol.

[R39] Sasaki J, Ishikawa K, Arita M, Taniguchi K (2012). ACBD3-mediated recruitment of PI4KB to picornavirus RNA replication sites. EMBO J.

[R40] Sbodio JI, Hicks SW, Simon D, Machamer CE (2006). GCP60 preferentially interacts with a caspase-generated golgin-160 fragment. J Biol Chem.

[R41] Sbodio JI, Machamer CE (2007). Identification of a redox-sensitive cysteine in GCP60 that regulates its interaction with golgin-160. J Biol Chem.

[R42] Shinoda Y, Fujita K, Saito S, Matsui H, Kanto Y, Nagaura Y, Fukunaga K, Tamura S, Kobayashi T (2012). Acyl-CoA binding domain containing 3 (ACBD3) recruits the protein phosphatase PPM1L to ER-Golgi membrane contact sites. FEBS Lett.

[R43] Smola M, Horova V, Boura E, Klima M (2020). Structural basis for hijacking of the host ACBD3 protein by bovine and porcine enteroviruses and kobuviruses. Arch Virol.

[R44] Sohda M, Misumi Y, Yamamoto A, Yano A, Nakamura N, Ikehara Y (2001). Identification and characterization of a novel Golgi protein, GCP60, that interacts with the integral membrane protein giantin. J Biol Chem.

[R45] Soupene E, Kuypers FA (2015). Ligand binding to the ACBD6 protein regulates the acyl-CoA transferase reactions in membranes. J Lipid Res.

[R46] Wang YJ, Wang J, Sun HQ, Martinez M, Sun YX, Macia E, Kirchhausen T, Albanesi JP, Roth MG, Yin HL (2003). Phosphatidylinositol 4 phosphate regulates targeting of clathrin adaptor AP-1 complexes to the Golgi. Cell.

[R47] Wang J, Sun H-Q, Macia E, Kirchhausen T, Watson H, Bonifacino JS, Yin HL (2007). PI4P promotes the recruitment of the GGA adaptor proteins to the trans-Golgi network and regulates their recognition of the ubiquitin sorting signal. Mol Biol Cell.

[R48] Waugh MG (2019). The Great Escape: how phosphatidylinositol 4-kinases and PI4P promote vesicle exit from the Golgi (and drive cancer). Bio-chem J.

[R49] Weixel KM, Blumental-Perry A, Watkins SC, Aridor M, Weisz OA (2005). Distinct Golgi populations of phosphatidylinositol 4-phosphate regulated by phosphatidylinositol 4-kinases. J Biol Chem.

[R50] Wong M, Munro S (2014). Membrane trafficking. The specificity of vesicle traffic to the Golgi is encoded in the golgin coiled-coil proteins. Science.

[R51] Wood CS, Schmitz KR, Bessman NJ, Setty TG, Ferguson KM, Burd CG (2009). PtdIns4P recognition by Vps74/GOLPH3 links PtdIns 4-kinase signaling to retrograde Golgi trafficking. J Cell Biol.

[R52] Yue X, Bao M, Christiano R, Li S, Mei J, Zhu L, Mao F, Yue Q, Zhang P, Jing S (2017). ACBD3 functions as a scaffold to organize the Golgi stacking proteins and a Rab33b-GAP. FEBS Lett.

[R53] Yue X, Qian Y, Zhu L, Gim B, Bao M, Jia J, Jing S, Wang Y, Tan C, Bottanelli F (2021). ACBD3 modulates KDEL receptor interaction with PKA for its trafficking via tubulovesicular carrier. BMC Biol.

