# Supplementary figures and images for: Recruitment of PI4KIIIβ to the Golgi by ACBD3 is dependent on an upstream pathway of a SNARE complex and golgins
Source: Mol Biol Cell. Author manuscript; Available in PMC 2024 Feb 1. (PMC7615549; doi:10.1091/mbc.E23-09-0376)

# Supplemental Materials

*Molecular Biology of the Cell*

Stalder *et al.*

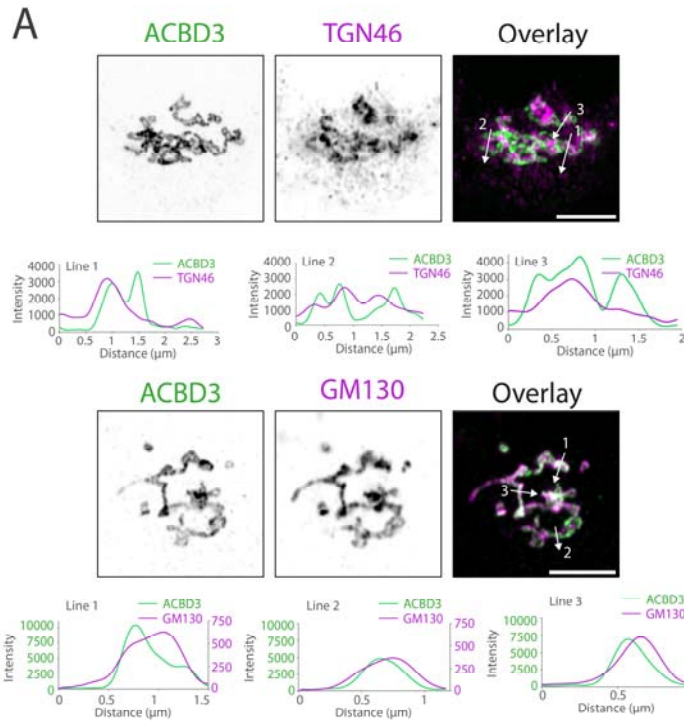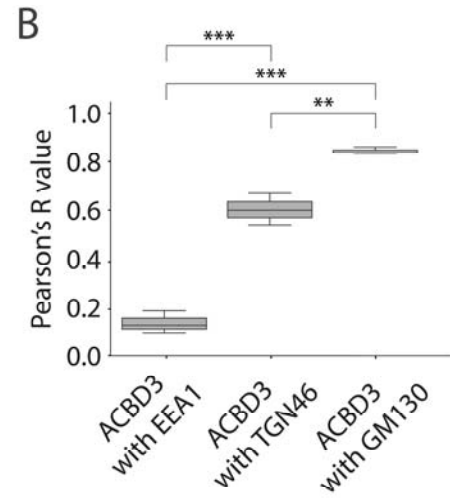

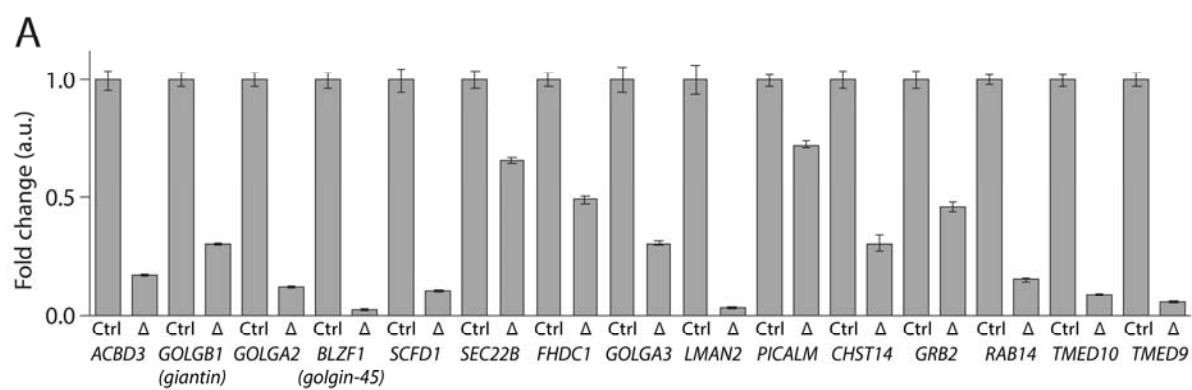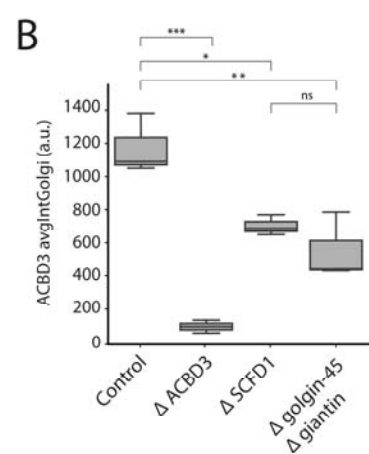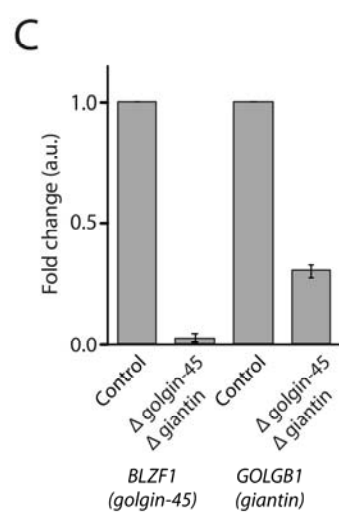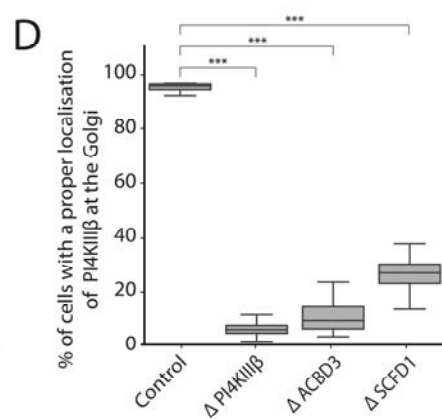

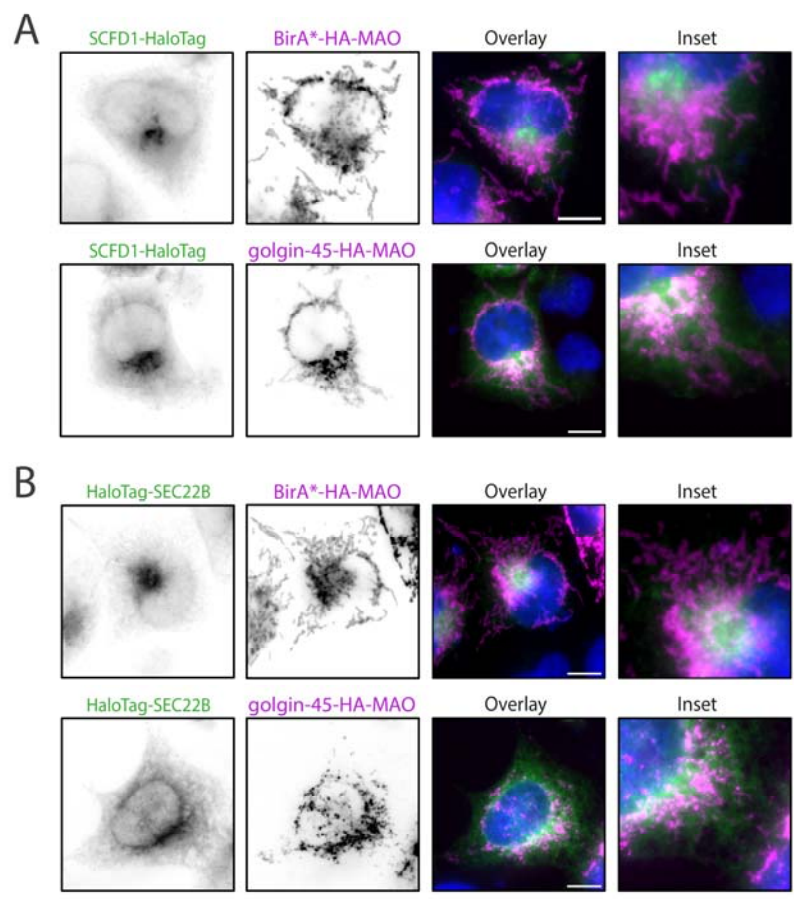

Supplement: Supplementary Information [file EMS193424-supplement-Supplementary_Information.pdf]
